# Prevalence of filaggrin gene polymorphisms (exon-3) in patients with atopic dermatitis in a multiracial Brazilian population^☆^

**DOI:** 10.1016/j.abd.2022.04.005

**Published:** 2023-01-18

**Authors:** Cristina Marta Maria Laczynski, Carlos D’Apparecida Santos Machado Filho, Hélio Amante Miot, Denise Maria Christofolini, Itatiana Ferreira Rodart, Paulo Ricardo Criado

**Affiliations:** a Discipline of Dermatology, Centro Universitário Faculdade de Medicina do ABC, Santo André, SP, Brazil; b Department of Dermatology, Faculty of Medicine, Universidade Estadual Paulista, Botucatu, SP, Brazil; c Department of Genetics, Centro Universitário FMABC, Santo André, SP, Brazil

Dear Editor,

Atopic dermatitis (AD) is a chronic, multifactorial disease whose clinical phenotype results from the interaction of genetic and environmental factors.^1^ Immune dysregulation and skin barrier integrity determine its severity, predisposing to infections and antigen permeability.^2^ It is a common reason for dermatological consultation, especially in childhood (<12 years) representing 25.8% of dermatological consultations.^3^

The gene encoding filaggrin (*FLG*) is highly polymorphic and is located in the region of the epidermal differentiation complex (1q21.3), encoding the most important proteins involved in skin barrier homeostasis. *FLG* is the main genetic factor associated with AD and its exon-3 transcribes most of the profilaggrin protein. Skin barrier alterations are present in AD patients without filaggrin changes; however, the presence of *FLG* variants leading to loss of function has been associated with clinical phenotypes such as persistent early-onset disease, asthma, and allergic sensitization.^1^, ^4^ Marked ethnic disparity has been observed in the frequency of *FLG* variants leading to loss of filaggrin function in AD.^5^

More than 60 variants in *FLG* leading to loss of filaggrin function have been identified in association with AD, with the most common among Europeans being c.1537C>T:R501X and 2282del4:S761Cfs*36 and, in sub-Saharan Africans, c.9947C>G:S3316*. Few studies have been carried out on Latin-American patients with AD. The objective of the present study was to assess the frequency of *FLG* variants (in exon-3) in AD patients to compare Brazilian and international populations and explore their clinical characteristics.

A cross-sectional study was carried out at the Dermatology Outpatient Clinic (FMABC; Santo André, São Paulo). Eighty patients with AD (Hanifin and Rajka’s criteria) of both sexes were included and examined by an experienced dermatologist to assess disease severity (SCORAD, EASI) and to collect venous blood samples for laboratory analysis and oral mucosa for genetic analysis. The participants/guardians signed a free and informed consent form.

The specimen collected for genetic analysis was obtained by swabbing the patients’ mucosa of the inner cheek and placing it in a sterile test tube (Oragene Collector OG-500®, DNA Genotek Inc., Kanata, Ontario). It was submitted to sequencing using the Sanger method.

DNA extraction was performed using ethanol precipitation and a prepIT 2P reagent provided together with the Oragene kit.

Polymerase chain reaction (PCR) and its sequencing analysis were performed focusing on exon-3 to identify the most common genetic variants– c.1537C>T:R501X (rs61816761) and c.2282del4:S761Cfs*36 (rs558269137) using validated primers from Thermo Fischer Scientific® (Applied Biosystems, Foster City, CA), (Hs00274028 forward: 5'CTA ACA CTG GAT CCC TGG TTC CTA 3' and reverse 5' CTG AGA CAG CAG AGC CAC CAA GA 3' and Hs00395823, forward: 5' CAG ACC TAT CTA CCG ATT GCT CGT 3' and reverse: 5' AAA TCA GGC ACTCGT CAC ACA CAG AA 3'). This strategy allowed the investigation of other variants in areas neighboring the target loci, but it did not cover the entire exon-3 coding region (Fig. 1). The PCR products were purified with DNA beads (Agencourt ‒ AMPure XP-Beckman Coulter, Brea, CA). The purified samples, together with 10 μL of these primers, were used for the sequencing reaction. The sequencing cycle was performed with the Big Dye Terminator v3.1 kit (Thermo Fisher Scientific). The sequencing products were submitted to capillary electrophoresis on the ABI 3500 DNA Analyzer (Applied Biosystems, Foster City, CA). The sequencing data were evaluated with Seq A (14) software (Applied Biosystems, Foster City, CA). PROVEAN (Protein Variation Effect Analyzer) v1.1 was used to predict whether a protein sequence variation caused by a missense substitution would affect protein function (available at https://provean.jcvi.org/index.php).

**Figure 1:**
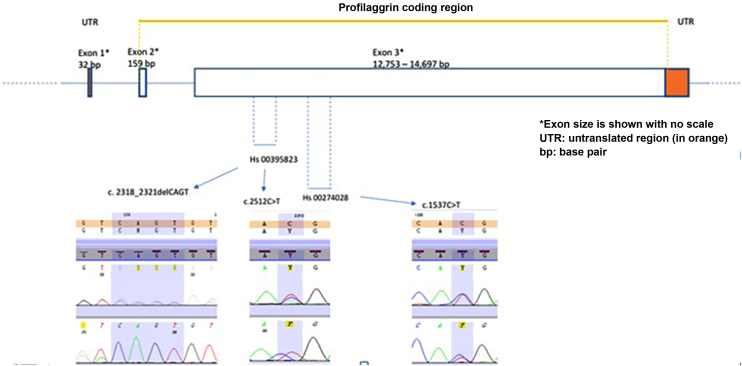
Schematic picture of the *FLG* gene including the distribution of exons, size of exons, introns, indication of profilaggrin coding region, position of primers and electropherograms of the three most frequent pathogenic variants found in the present sample.

The prevalence of each identified variant was compared to the Brazilian public database of genomic variants (ABraOm; hg38 – https://abraom.ib.usp.br/) with 1,171 samples from the population of the same region and an international database (gnomAD; v3.1.2 – https://gnomad.broadinstitute.org/) with 76,156 unrelated individuals of different ethnicities. The statistical significance was set at p ≤ 0.001.^6^

The main demographic data, clinical severity, eosinophilia, and IgE levels are shown in Table 1. Most patients had moderate and severe AD (79%), elevated IgE levels (98%), and eosinophilia (68%).

**Table 1:** Main clinical, demographic and laboratory characteristics of the Brazilian AD sample (n = 80).

Variables		Values
Sex, n (%)	Female	40 (50)
	Male	40 (50)
Age (years), mean (SD)		16 (12)
Phototype, n (%)	I‒II	24 (30)
	III‒IV	51 (64)
	V‒VI	5 (6)
Ethnicity, n (%)	Caucasian	54 (68)
	Asian	1 (1)
	Afro-descendant	25 (31)
AD severity, n (%)	Mild	17 (21)
	Moderate	45 (56)
	Severe	18 (23)
SCORAD, mean (SD)		36 (17)
EASI, mean (SD)		13.5 (10.2)
Elevated levels of IgE^a^, n (%)		79 (98)
Eosinophils > 5%, n (%)		46 (68)

AD, Atopic dermatitis; IgE, Serum immunoglobulin E; SD, Standard deviation; SCORAD, Scoring Atopic Dermatitis; EASI, Eczema Area Severity Index.

a According to the reference values for each age group: 0‒1 year: up to 15.0 IU/mL; 1‒2 years: 1.0 to 19.0 IU/mL; 2‒3 years: up to 32 IU/mL; 4‒9 years: up to 101.0 IU/mL; over 15 years: 1.0 to 183.0 IU/mL; Median: 98.44.

The results of the genetic analysis of the AD sample and the comparison with the two population controls are shown in Table 2. Twenty-six *FLG* exon-3 genetic variants were detected in 60 patients (75%; 95% CI 65%‒85%). Homozygosity and compound heterozygosity were not identified.

**Table 2:** FLG gene variants (exon 3) in 80 Brazilian patients with atopic dermatitis and their prevalence according to AbraOM population databases (from the same Brazilian population) and gnomAD (from Latin, African-American, European and international populations).

Variant	Zygosity	dbSNP	Protein consequence	AD, n (%)	AbraOM (Brazil)	gnomAD			
						Latin/ Mixed	European	African-American	International
c.1396A>G	HT	rs2011331	T454A	38 (47.50)	33.09%	39.36%	16.02%	47.84%	30.25%
c.1468C>T	HT	rs11584340	P478S	39 (48.75)	25.06%	35.76%	15.90%	13.81%	20.34%
c.1476T>C	HT	rs561848191	Synonymous	2 (2.50)	<0.01%	0.01%	<0.01%	0.53%	0.14%
c.1521G>A	HT	rs75530805	Synonymous	1 (1.25)	NF	<0.01%	<0.01%	<0.01%	<0.01%
c.1521G>C	HT	rs75530805	Synonymous	21 (26.25)	1.49%	0.60%	<0.01%	5.1%	1.47%
c.1530G>C	HT	rs13376095	E498D	3 (3.75)	0.05%	0.22%	<0.01%	2.62%	0.74%
c.1537C>T	HT	rs61816761	R501X^a^	3 (3.75)	0.08%	<0.01%	<0.01%	<0.01%	<0.01%
c.1591C>A	HT	rs12036682	H519N	1 (1.25)	0.03%	0.05%	0.06%	<0.01%	0.58%
c.1665T>G	HT	rs152285733	Synonymous	8 (10.00)	NF	NF	NF	NF	NF
c.1712A>G	HT	rs546475787	H559R	6 (7.50)	<0.01%	<0.01%	<0.01%	<0.01%	<0.01%
c.1737A>C	HT	rs71625187	Synonymous	25 (31.25)	NF	<0.01%	<0.01%	<0.01%	<0.01%
c.1777A>T	HT	rs145627745	T581S	31 (38.75)	0.09%	0.49%	1.37%	0.27%	0.79%
c.1800T>C	HT	rs152285598	Synonymous	14 (17.50)	NF	NF	NF	NF	NF
c.2167C>T	HT	rs115087788	R711C	1 (1.25)	<0.01%	0.20%	<0.01%	0.82%	0.25%
c.2210C>T	HT	rs3120655	T725I	13 (16.25)	8.07%	3.53%	0.17%	34.83%	9.98%
c.2217C>A	HT	rs7512779	H727Q	2 (2.50)	1.96%	<0.01%	<0.01%	<0.01%	<0.01%
c.2261C>A	HT	rs3120654	S742Y	13 (16.25)	8.03%	3.47%	0.13%	34.77%	10.06%
c.2271T>C	HT	rs150144110	s745=	1 (1.25)	0.01%	<0.01%	<0.01%	0.19%	<0.01%
c.2299G>A	HT	rs74129461	E755K	23 (28.75)	23.40%	34.96%	15.90%	7.73%	18.57%
c.2282del4CAGT#	HT	rs558269137	S761Cfs*36#	3 (3.75)	0.06%	<0.01%	2.19%	<0.01%	1.26%
c.2319A>C	HT	rs11204979	Synonymous	2 (2.50)	0.82%	0.59%	<0.01%	5.41%	1.57%
c.2377G>C	HT	rs148739675	D781H	1 (1.25)	0.21%	0.07%	<0.01%	0.66%	0.19%
c.2396C>T	HT	rs77032592	S787F	1 (1.25)	0.56%	0.28%	<0.01%	3.26%	0.94%
c.2512C>T	HT	rs115746363	R826X^a^	2 (2.50)	0.13%	<0.01%	<0.01%	0.72%	0.21%
c.2544T>C	HM	rs3120653	Synonymous	37(46.25)	33.60%	<0.01%	<0.01%	0.02%	<0.01%

dbSNP (rs), Reference SNP number in international databases (chromosomal position); HT, Heterozygous; HM, Homozygous; AD, Atopic dermatitis; NF, Not found.

a Mutation with loss of function (pathogenic).

*FLG* variants with filaggrin loss-of-function were observed in eight AD patients (10%; 95%CI 3%–17%). Two variants prevalent in the world (c.1537C>T:R501X and 2282delCAGT:S761Cfs*36) were observed in six (7.5%) patients. Another pathogenic variant (c.2512C>T:R826X) was identified in two (2.5%) patients. These three pathogenic variants were more prevalent in the AD sample than in the Brazilian controls (p < 0.001). The 2282delCAGT:S761Cfs*36 variant is common among Europeans, while c.2512C>T:R826X is common among African-Americans.

Eight synonymous variants were found in the *FLG* gene, four of them widely distributed in Brazil and worldwide. However, four of them (c.1665T>G:rs152285733, c.1737A>C: synonymous, c.1521G>A: synonymous, and c.1800T>C: synonymous) were observed to be more common in the AD sample than in the world (p < 0.001). The variant c.1476T>C: synonymous, rare in the Brazilian and in the international population (p < 0.001), is common among African-Americans. The synonymous c.2544T>C, considered common among AD patients and regional controls, is extremely rare in the world (p < 0.001).

Fourteen missense variants were detected, with two of them (c.1712A>G:H559R, c.1777A>T:T581S) being more common among AD patients than in regional and international controls (p < 0.001).

No *FLG* variant was associated with AD clinical severity, eosinophilia, or elevated serum IgE levels (p > 0.1).

These results reinforce the high polymorphism of exon-3 in *FLG* and its ethnic association, making it difficult to generalize genomic results in relation to AD phenotypes, especially in highly mixed populations.^5^, ^7^, ^8^

According to the literature, null variants were expected in 14% to 42% of AD patients, and, of these, 20 were found in exon-3. In addition, variations in *FLG* have been reported among AD patients of different ethnicities.^8^

The Brazilian population has a multiracial composition, after 500 years of miscegenation amongst individuals from Western Europe, Africa, and the Amerindian populations.^9^ The c.2512C>T:R826X and 2282delCAGT:S761Cfs*36 variants in AD patients corroborate these ancestries, meanwhile the c.2544T>C: synonymous variant is characteristic of this region.

Considering the epidemiological association with disease development, isolated variants in *FLG* gene do not fully explain the variation in AD severity, eosinophilia, or elevated IgE levels, reinforcing the multifactorial aspects of the disease.^1^, ^8^, ^10^

In conclusion, we authors identified null variants of the *FLG* gene (in exon-3) (c.1537C>T:501X, c.2282del4:S761Cfs*36 and c.2512C>T:R826X) in 10% of Brazilian patients with AD, but without an association with the main clinical characteristics of AD.

## Financial support

Fundo de Apoio à Dermatologia do Estado de São Paulo ‒ Sebastião Sampaio (FUNADERSP).

## Authors’ contributions

Cristina Marta Maria Laczynski: Design and planning of the study, collection, analysis and interpretation of data; approved the final version of the manuscript.

Carlos D’Apparecida Santos Machado Filho: Critical review of the intellectual content; approved the final version of the manuscript.

Hélio Amante Miot: Critical review of the intellectual content; approved the final version of the manuscript.

Denise Maria Christofolini: Critical review of the intellectual content; approved the final version of the manuscript.

Itatiana Ferreira Rodart: Processing of genetic material, analysis and interpretation of data and review of the intellectual content; approved the final version of the manuscript.

Paulo Ricardo Coelho: Critical review of the intellectual content; approved the final version of the manuscript.

## Conflicts of interest

None declared.
